# Attentional Patterns Toward Pain-Related Information: Comparison Between Chronic Pain Patients and Non-pain Control Group

**DOI:** 10.3389/fpsyg.2020.01990

**Published:** 2020-08-05

**Authors:** Jieun Lee, Suk-Won Ahn, Amy Wachholtz, Jang-Han Lee

**Affiliations:** ^1^Department of Psychology, Chung-Ang University, Seoul, South Korea; ^2^Department of Neurology, College of Medicine, Chung-Ang University, Seoul, South Korea; ^3^Department of Psychology, University of Colorado Denver, Denver, CO, United States; ^4^Department of Psychology, Chung-Ang University, Seoul, South Korea

**Keywords:** attentional patterns, pain-related information, chronic pain patients, non-pain control group, pain catastrophizing

## Abstract

Although the evidence for attentional bias to pain-related information among individuals with chronic pain has been well established, there are a number of inconsistencies in the research that have been observed due to sample characteristics. Therefore, the present study expanded upon previous studies by including patients with a variety of chronic pain conditions and compared a chronic pain patient sample with healthy community sample. We also investigated how pain catastrophizing and other psychological factors in chronic pain patients affected attentional patterns to pain-related information. Forty chronic pain patients from the departments of neurology and rheumatology of an academic medical center hospital and 40 participants without chronic pain from a university that is located in Seoul, South Korea were recruited for the present study. Patients observed pictures of faces displaying pain that were presented simultaneously with faces with neutral expressions, while their eye movements were measured using an eye-tracking system. Independent *t*-tests were conducted to investigate attentional preferences to pain stimuli between the chronic pain and control groups. No significant attentional differences in pain-neutral pairs were found for both chronic pain and control group. A one-way MANOVA was conducted to examine the role of pain catastrophizing on psychological factors and attentional engagement to pain stimuli. No significant results for the attentional bias to pain stimuli among chronic pain patients may indicate that chronic pain patients who have suffered from chronic pain for a long time and have been treated for their chronic pain in the hospital may interpret pain-related information not as threatening. Clinical implications related to use in pain treatment and future research suggestions are discussed.

## Introduction

The evidence for the attentional bias to pain-related information for acute or experimental pain has been well established. However, results regarding attentional bias to pain-related information among individuals with chronic pain have been inconsistent. A previous attentional bias meta-analysis (Gaffiero et al., 2019) found relatively consistent evidence for the attentional bias with word stimuli but inconsistent results for an attentional bias for facial expression stimuli. Some studies (Vervoort et al., 2013; Lee et al., 2018) found evidence for an attentional bias to pain facial expression stimuli among individuals with chronic pain whereas others (Lee et al., 2019; Mazidi et al., 2019) did not find significant results even though these studies mentioned above utilized the similar eye tracking methodology (i.e., utilizing facial expression photos sourced from the database such as Montreal database and KUFEC, and pairing pain facial expressions with neutral facial expressions while their eye movement were measured by an eye tracker).

Recent attentional bias research has been guided by the threat-interpretation model (Todd et al., 2015), which suggests that the degree to which individuals interpret stimuli as threatening influences attentional patterns toward threat-related information differently in different stages of attentional processes. For instance, during the early stage of attention, interpretation bias increases attentional focus to threat-related information. For the later stage of attention, interpretation bias moderates the attention focus, low interpretation bias leads to easy disengagement from threat-related information whereas high levels of interpretation bias lead to attentional avoidance from threat-related information. This model may partially explain the inconsistent results in published attentional bias studies. Eye tracking methodology (i.e., eye movements during the gaze toward photo stimuli are measured by the camera attached below the computer screen while the participants freely view photos presented on the computer screen; Lee et al., 2018) can capture the entire attentional process including early and late stages of attention unlike the dot probe task (i.e., the participants view the vertically presented pairs of word stimuli presented during a certain period of time and the subsequent visual probe such as a fixation cross located either the upper or lower location to replace previous word stimuli. Then, participants indicate the location of the probe by pressing a computer key as quickly as possible and their response time is measured; Schoth et al., 2019), so eye tracking methodology can be used in attentional bias studies to obtain preliminary data for the threat interpretation model (Gaffiero et al., 2019).

Several recent eye tracking studies that investigated attentional bias to pain facial expression stimuli found that cognitive factors of pain (i.e., attention, interpretation) interact with psychological factors in the context of chronic pain experience. Pain catastrophizing is defined as a thinking style which over-estimates the potential risks associated with potential or actual pain. Pain catastrophizing is associated with higher levels of pain severity, psychological distress, maladaptive coping, and increased disability (Kadimpati et al., 2015). Previous research (Heathcote et al., 2015) found that adolescents with high levels of pain catastrophizing were more likely to interpret ambiguous stimuli as more threatening. Studies (Vervoort et al., 2013; Lee et al., 2018) that investigated adults with chronic pain in the community found that pain catastrophizing increases attentional bias to pain facial expressions. In contrast, another study (Lee et al., 2019) found no significant effect of pain catastrophizing on attentional bias to pain facial expressions; however, the authors argued that their study assessed a unique population of chronic pain patients who received hospital-based care for their chronic pain. They argued that experiencing a level of pain, and severity that required hospitalization, as well as exposure to intensive chronic pain treatment might have altered the participants’ responses toward pain facial expressions.

Thus, previous studies included community-based adult samples with chronic pain, or chronic pain patient samples with a particular disorder (i.e., musculoskeletal disorders). Therefore, the current study expanded upon previous studies by including patients with a variety of chronic pain conditions (i.e., musculoskeletal disorders, autoimmune diseases, other type of non-cancer pain) and compared a chronic pain patient sample with healthy community sample.

Expanding on previous research, the present study would investigate the effects of pain catastrophizing on the attentional preference to pain facial expressions. By utilizing eye tracking methodology, we would examine how pain catastrophizing levels can influence attentional preferences to pain facial expressions throughout the time-course of attention. The current study would also investigate similarities and differences in the attentional patterns to pain facial expressions between a chronic pain patient group and a non-pain control group in order to determine whether attentional bias to pain facial expressions is specific to chronic pain patients. The present study hypothesized: (1) Chronic pain patients would show attentional preference to pain facial expressions compared to neutral expressions whereas there would be no significant differences in attentional engagement found between pain and neutral facial expressions within the control group; and (2) Level of pain catastrophizing would result in different attentional patterns at different stages of attention – individuals with a high pain catastrophizing level would initially gravitate toward pain-related information during the early stage of attention and would demonstrate attentional avoidance from pain-related information during the later stage of attention.

## Materials and Methods

### Participants

Forty chronic pain patients were recruited from the departments of neurology and rheumatology at an academic medical center hospital in Seoul, South Korea. Inclusion criteria for the chronic pain group were: (1) previously diagnosed with a chronic pain disorder by a physician (2) aged 19 years or over; and (3) having normal or corrected-to-normal vision. Exclusion criteria were: (1) inability to read Korean (2) not being able to view pictures presented on the screen 60 cm away clearly without wearing eyeglasses (3) having had LASIK or LASEK surgery, and (4) having cataract(s) or glaucoma. The mean age of chronic pain patients was 44.78 years (SD = 14.68) and consisted of 67.5% female. 40 participants in control group were recruited from a university in Seoul and their demographic characteristics (i.e., age, gender) were matched with the chronic pain patients. Inclusion criteria for the healthy control group were: (1) aged 19 years or over and (2) having normal or corrected-to-normal vision. Exclusion criteria were: (1) suffering from any form of chronic or recurrent pain. The study was approved by the Institutional Review Board of Chung-Ang University Hospital (1711-002-267).

### Measures

#### Pain Intensity

Pain Intensity Questions (Lee et al., 2018, 2019) includes 3 items which assess (1) the degree of pain one currently feels, (2) the pain during the past week, and (3) the pain during the past 3 months. Participants rated on a 11-point scale, ranging from 0 to 10 Pain Facial Expression Scale. The internal consistency of this study was 0.96.

#### Pain Disability

The Pain Disability Index (PDI) that was originally developed by Pollard (1984) and translated by Hong (2010) was used (Pollard, 1984; Hong, 2010). PDI is a questionnaire that measure the degree of subjective disruption experienced due to chronic pain in seven different domains of life (e.g., home, social, recreational, occupational, sexual life, self-management, and life-support). For each life domain, participants were asked rate to provide disability ratings on 11-point scales from 0 (no disability) to 10 (total disability). The higher score represents pain interfering more with life. The internal consistency for the present study was 0.94.

#### Pain Catastrophizing

Pain Catastrophizing Scale (PCS) is a 13 item scale measuring three aspects of pain catastrophizing (Sullivan et al., 1995). This measure consists of three subscales of pain catastrophizing: rumination (e.g., “I can’t seem to keep it out of my mind”); magnification (e.g., “I wonder whether something serious may happen”); and helplessness (e.g., “There is nothing I can do to reduce the intensity of pain”). Participants were asked to rate their responses on a five-point Likert scale ranging from 0 (not at all) to 4 (always). This measure has been extensively used in chronic pain researches, and has shown high internal consistency (α = 0.87) and validity in clinical and experimental groups (Sullivan et al., 1995). This scale has been translated and standardized in Korean population (Cho et al., 2013). Cronbach’s alpha of K-PCS was 0.93 (Cho et al., 2013) and 0.91 for our previous study (Lee et al., 2018). The internal consistency of this study was 0.94.

#### Psychological Flexibility

Acceptance Action Questionnaire-II (AAQ-II) is a 10 items scale on 7 points Likert scale (Bond et al., 2011). This scale ranged from (1: not at all) to (7:always). The higher the score, the greater the degree of psychological flexibility. The present study utilized Korean version of AAQ-II (Heo et al., 2009). The internal consistency was 0.85 (Heo et al., 2009) and that of the present study was 0.89.

#### Anxiety and Depression

The Hospital Anxiety Depression Scale (HADS) is a 14-item questionnaire that is designed to assess state anxiety and depression in clinical populations with physical illness (Zigmond and Snaith, 1983). The HADS is an overall measure of emotional distress which is consisted of two subscales such as anxiety and depression. These two subscales contain four-point ranging from 0 (not at all) to 3 (all the time) Likert scale for each item. Total scores range from 0 representing no anxiety or depression to 21 representing high levels of anxiety and depression. A Korean language version of the HADS (K-HADS) has also shown good reliability and validity estimates in clinical samples (Oh et al., 1999). The values of Cronbach’s alpha coefficient of anxiety and depression were 0.89 and 0.86, respectively. The internal consistency of anxiety and depression for the present study were 0.88 and 0.76.

### Stimulus Materials

The stimulus set consisted of pictures of 8 adult faces (4 male and 4 female) which were obtained from our previous study on college students (Lee et al., 2018). Those photos were originally obtained by the Korea University Collection – KUFEC (Lee et al., 2006). 32 pictures of pain expression (4 pictures per 1 adult face) were created for our previous study (Lee et al., 2018) and were included in the present study. All images obtained by KUFFC were converted into monochrome images in order to decrease the effects of emotions associated with color (Priebe et al., 2015). Furthermore, in order to diminish distractions associated with hairstyles, all images were cropped to show only faces (Vervoort et al., 2013). After we made these adjustments, all photos displaying painful facial expressions were validated by 15 psychology graduate student judges (5 males, 10 females) and 45 undergraduate student judges (26 males, 19 females) as part of our previous study that investigated attentional bias to pain-related information among individuals with chronic pain (Lee et al., 2018). For instance, the mean of painful facial expression (*M* = 5.67, SD = 0.51) was significantly different from neutral facial expression (*M* = 2.11, SD = 0.41; *t* (22) = −20.346, *p* < 0.001).

Using these pictures, we created 16 pairs of pictures; two pictures (pain, neutral) were horizontally aligned in one slide. Pain and neutral pairs were presented twice, and the locations of those photos were switched during the second viewing in order to avoid the location bias (Lee et al., 2018, 2019).

### Free-Viewing Task and Apparatus

In the free-viewing task, participants were seated in front of the monitor and asked to hold their posture to minimize head movement, and gaze at the picture stimuli through a monitor which was placed 60 cm away. Eye movements were calibrated and then stimulus presentation was in order of the fixation cross (1,000 ms), facial expression stimulus (3,000 ms), and blank screen (1,000 ms). The task consisted of practice trials and experimental trials. Before starting the experimental trials, participants learned how to freely look at the pictures presented on the screen during practice trials. The stimulus paradigm for the measurement of attentional bias was based on the previous study using eye movement tracking equipment (Lee et al., 2018). The size of each photo stimuli appeared on the monitor screen was 55 cm (width) × 24 cm (length) with a resolution of 1920 × 1080 pixels, and the viewing angle was 38°.

Tobii TX300 eye-tracker (Tobii Technology, Stockholm, Sweden) was utilized to measure eye movements. This eye tracker has an LCD 22 inch monitor and a camera to track eye movements is located at the bottom of the screen. TX300 has 300 Hz sampling rate and the 65 cm (27”) distance. Fixation is defined when participants’ gaze is remained on defined AOI (area of interest), which was the entire photo, for at least 100 ms.

### Statistical Analysis

In order to investigate similarities and differences for initial attention and maintained attention between chronic pain group and non-pain control group, initial fixation duration and total gaze duration for pain stimuli were compared with those of neutral stimuli by performing *t*-tests for each group (chronic pain group vs. non-pain control group). Repeated measures analyses of variance (ANOVAs) with the within-factors “time” (0–500 ms vs. 500–1000 ms vs. 1000–1500 ms vs. 1500–2000 ms vs. 2000–2500 ms vs. 2500–3000 ms) and “stimulus type” (pain vs. neutral) were conducted for high pain catastrophizing group and low pain catastrophizing group to investigate differences in the time-course of maintained attentional patterns toward pain stimuli. Lastly, to examine the effects of pain catastrophizing on attentional bias to pain for chronic pain group, chronic pain patients were divided into two groups (high pain catastrophizing vs. low pain catastrophizing) depending on their pain catastrophizing level by using median split method (Iacobucci et al., 2015). One-way MANOVA was conducted to examine the role of pain catastrophizing (high pain catastrophizing group vs. low pain catastrophizing group) on psychological factors and attentional engagement to pain stimuli.

The Shapiro-Wilk test was used to examine normal distribution. The values of the Shapiro-Wilk test for all outcomes by two groups (chronic pain group vs. control group) were greater than 0.05, indicating that the data was normally distributed. For the control group, Shapiro-Wilk test for total gaze duration to pain was non-significant which indicates normal distribution of the data (*W* = 0.974, df = 31, *p* = 0.621) as was the total gaze duration to neutral stimuli (*W* = 0.975, df = 31, *p* = 0.681). For the chronic pain group, we used the Shapiro-Wilk test to examine different catastrophizing groups. The total gaze duration to pain for the low catastrophizing group was *W* = 0.950, df = 20, *p* = 0.374 and for the high catastrophizing group was *W* = 0.940, df = 15, *p* = 0.379. The total gaze duration for neutral stimuli in the low catastrophizing group was *W* = 0.929, df = 20, *p* = 0.145, and total gaze duration for neutral stimuli in the high catastrophizing group was *W* = 0.945, df = 15, *p* = 0.445.

## Results

Table 1 displays the descriptive statistics of demographic information (i.e., age, gender, drinking, smoking), psychological factors (i.e., pain catastrophizing, psychological flexibility, depression, anxiety), and participants’ pain information. There were no significant differences between groups on the demographic variables. The chronic pain group reported significantly more current pain, pain catastrophizing, depression, and anxiety compared to the control group. In contrast, the control group reported significantly higher levels of psychological flexibility compared to chronic pain group.

**TABLE 1:** Descriptive statistics of demographic information and psychological variables by two groups.

	Mean (SD)			
Variables	Chronic pain group (*n* = 35)	Control group (*n* = 31)	*F/*χ^2^	*p*
Age	44.67 (14.86)	47.61 (15.92)	0.637	0.427
Gender			0.25	0.875
Male	30.08%	29.00%		
Female	69.20%	71.00%		
Smoking			1.362	0.243
Yes	15.40%	6.5%		
No	84.60%	93.5%		
Drinking			0.006	0.939
Yes	41.00%	41.90%		
No	59.00%	58.10%		
3 month pain	4.56	0.06	81.027	0.000
Type of chronic pain				
Musculoskeletal disorders	9 (25%)	0		
Autoimmune diseases	21 (60%)	0		
Others	5 (14%)	0		
Pain catastrophizing	18.03 (9.70)	4.19 (5.59)	49.80	0.000
Psychological flexibility	48.77 (11.93)	55.32 (10.46)	5.80	0.019
Depression	14.51 (3.72)	11.10 (2.90)	17.60	0.000
Anxiety	13.64 (4.07)	10.97 (2.29)	10.68	0.002

As shown in Table 2, the chronic pain group initially gravitated toward pain stimuli and the opposite pattern (initial attentional preference to neutral stimuli) was identified in the control group although those differences were not statistically significant. There were no significant differences in maintained attention between pain and neutral stimuli across the chronic pain and control groups.

**TABLE 2:** Means/SD/*t*-test results of the initial fixation and total gaze durations by groups.

	Expression		*t*	*p*	Cohen’s *d*
Initial fixation durations	Pain M (SD)	Neutral M (SD)			
Chronic Pain Group	0.2559 (0.154)	0.2328(0.124)	−0.684	0.498	0.165
Control Group	0.2177 (0.104)	0.2632(0.138)	1.500	0.144	0.372

**Total gaze durations**	**Pain M (SD)**	**Neutral M (SD)**	***t***	***p***	

Chronic Pain Group	0.9213 (0.166)	0.9197 (0.145)	−0.090	0.928	0.009
Control Group	0.9568 (0.149)	0.9513 (0.157)	−0.034	0.739	0.036

Shown in Table 3, repeated measures ANOVAs were conducted with “time” (0–500 ms vs. 500–1000 ms vs. 1000–1500 ms vs. 1500–2000 ms vs. 2000–2500 ms vs. 2500–3000 ms) and “stimulus type” (neutral vs. pain) for both high and low catastrophizing groups. Significant effects were found on time only for high catastrophizing group [*F*(5,70) = 8.469, *p* < 0.01, η2 = 0.809] and low catastrophizing group [*F*(5,95) = 12.686, *p* < 0.001, η2 = 0.809].

**TABLE 3:** Summary of repeated measure ANOVA for two groups.

High P-CAT group	*F*	*p*	η^2^
Time	8.469	0.002**	0.809
Stimulus type	0.005	0.943	0.000
Time × Stimulus type	0.656	0.665	0.247
**Low P-CAT group**	***F***	***p***	**η^2^**
Time	12.686	0.000***	0.809
Stimulus type	0.007	0.934	0.000
Time × Stimulus type	1.139	0.383	0.275

**p < 0.01, ***p < 0.001. Stimulus type: pain vs. neutral. P-CAT, pain catastrophizing level.

**FIGURE 1:**
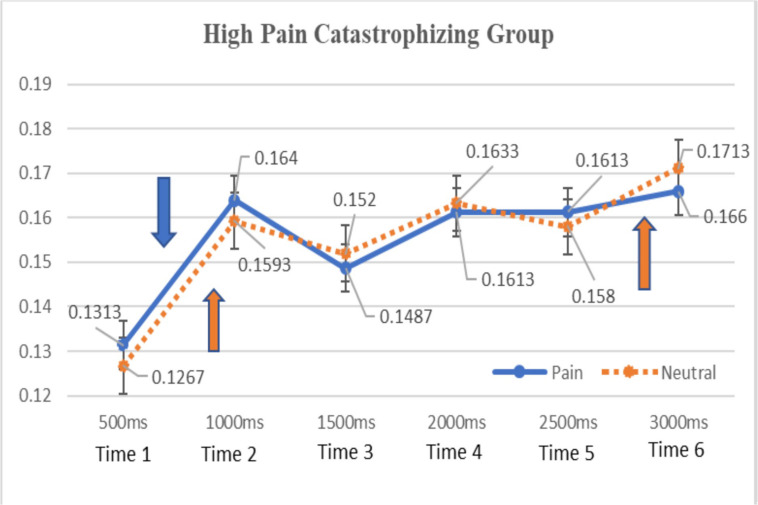
Means of total gaze durations to pain and neutral facial stimuli by time (high pain catastrophizing group). Means of total gaze durations for pain and neutral stimuli were labeled and standard deviation bars were also included for each time point. Down-arrow symbol = a significant difference in gaze duration of pain stimuli between time 1 and 2. Up-arrow symbols = significant differences in gaze durations of neutral stimuli in time 1–2 and time 5–6. No significant differences were found between pain and neutral stimuli for all time point.

**FIGURE 2:**
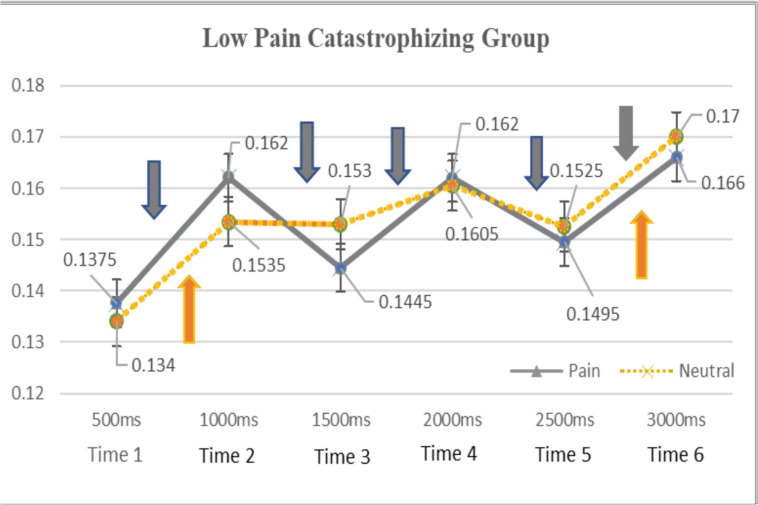
Means of total gaze durations to pain and neutral facial stimuli by time (low pain catastrophizing group). Means of total gaze durations for pain and neutral stimuli were labeled and standard deviation bars were also included for each time point. Down-arrow symbol = significant differences in gaze durations of pain stimuli between time 1–2, time 2–3, time 3–4, time 4–5, time 5–6. Up-arrow symbols = significant differences in gaze durations of neutral stimuli between time 1–2 and time 5–6. No significant differences were found between pain and neutral stimuli for all time point.

As shown in Figure 1, for the low pain catastrophizing group, gaze durations to pain-stimuli were greater than those for neutral-stimuli during the early stage of attention (0–1000 ms) and the opposite attentional patterns (attentional preference to neutral-stimuli as opposed to pain-stimuli) was found for the remaining attentional process (i.e., from 1000ms to 3000 ms). Similar attentional patterns (see Figure 2) were observed for the high pain catastrophizing group except there was maintained attention to pain-stimuli, which was observed during the later stages of attention (i.e., from 2000–3000 ms).

As shown in Figures 1, 2, the present study investigated the time-course of attention across 3000 ms and found that chronic pain patients, regardless of their pain catastrophizing levels, equally increased their attention to both pain and neutral stimuli across time. Specifically, the high pain catastrophizing group had significantly longer gaze durations toward pain stimuli during the 500–1000 ms period, shorter during the 1000–1500 ms period, and gradually increasing during the rest of the time periods. The gaze durations to neutral stimuli followed the similar course of attention as those of pain stimuli except there was a drop in gaze durations between 2000–2500 ms. For the low pain catastrophizing group, the gaze durations toward both pain and neutral stimuli rapidly increased during the 500ms–1000 ms period, fluctuated during the 1000–2500 ms period, and increased during the 2500–3000 ms period.

Table 4 showed that the high pain catastrophizing group initially preferred neutral stimuli while the low pain catastrophizing group initially gravitated toward pain stimuli. The opposite attentional patterns (e.g., attentional preference to pain stimuli for the high pain catastrophizing group and attentional preference to neutral stimuli for the low pain catastrophizing group) were found for the total gaze durations. However, all differences between pain and neutral stimuli were not statistically significant.

**TABLE 4:** Means/SD and *t*-test results of the initial fixation and total gaze durations by groups.

	Expression		*t*	*p*	Cohen’s *d*
Initial fixation durations	Pain M (SD)	Neutral M (SD)			
High P-CAT	0.2180 (0.0956)	0.2400 (0.1078)	−0.640	0.532	0.215
Low P-CAT	0.2970 (0.1899)	0.2225 (0.1350)	1.313	0.205	0.449

**Total gaze durations**	**Pain M (SD)**	**Neutral M (SD)**	***t***	***p***	**Cohen’s *d***

High P-CAT	0.9327 (0.1067)	0.9307 (0.1310)	0.073	0.943	0.017
Low P-CAT	0.9215 (0.2039)	0.9235 (0.1623)	−0.083	0.934	0.011

*P-CAT, pain catastrophizing level.*

A one-way MANOVA was conducted on the chronic pain group to examine the role of pain catastrophizing (high pain catastrophizing group vs. low pain catastrophizing group) on psychological factors and attentional engagement to pain stimuli. The result (as shown in Table 5) indicated that significant differences were found on pain disability (*F*(1,33) = 55.104, *p* < 0.001, η2 = 0.625) and depression (*F*(1,33) = 6.844, *p* < 0.05, η2 = 0.172) but no significant differences on 3 month pain levels (*F*(1,33) = 4.096, *p* = 0.51, η2 = 0.110), psychological flexibility (*F*(1,33) = 0.227, *p* = 0.637, η2 = 0.007) and anxiety (*F*(1,33) = 1.523, *p* = 0.226, η2 = 0.044). These results suggested that chronic pain patients with high levels of pain catastrophizing reported high levels of current pain, pain disability and depression compared to those with low levels of pain catastrophizing. Additionally, in the present study, chronic pain patients with high pain catastrophizing levels attended to pain stimuli more than neutral stimuli whereas the opposite pattern (attentional preference to neutral stimuli) occurred for low pain catastrophizing group, however, differences were not significant [*F*(1,33) = 0.037, *p* = 0.848, η2 = 0.001].

**TABLE 5:** Mean/SD of psychological factors and total gaze durations by two pain catastrophizing groups.

	*M (SD)*	*M (SD)*	*F*	*p*	η^2^
	H-PC (*N* = 15)	L-PC (*N* = 20)			
3 Month pain level	6 (2.204)	4.4 (2.393)	4.096	0.051	0.110
Pain disability	36.07 (14.94)	20 (13.86)	10.777	0.002**	0.246
Depression	16.67 (3.66)	13.85 (2.72)	6.844	0.013*	0.172
Anxiety	15.07 (4.46)	13.4 (3.53)	1.523	0.226	0.044
Psychological flexibility	46.87 (12.55)	48.8 (11.37)	0.227	0.637	0.007
Total GD for pain	0.9327 (0.1067)	0.9215 (0.20394)	0.037	0.848	0.001
Total GD for neutral	0.9307 (0.1310)	0.9235 (0.1623)	0.020	0.889	0.001

* p < 0.05, ** p < 0.01; H-PC, high pain catastrophizing group; L-PC, low pain catastrophizing group; GD, gaze duration.

## Discussion

The present study examined attentional preference to pain facial expressions, which were paired with neutral facial expressions for both chronic pain patients and healthy adults using eye tracking methodology. The result of the current study did not support the evidence for the attentional bias to pain facial expressions among chronic pain patient group. Previous research has found consistent evidence for the attentional bias to pain words but not for facial expression stimuli (Lee et al., 2019; Mazidi et al., 2019). It may be that facial expression stimuli are not as relevant to chronic pain patients’ pain experiences as photos of affected areas or pain word stimuli. Facial expression stimuli may not be as direct reference to their pain experience, but chronic pain patients may consciously recognize and apply pain-related words to their pain experiences.

Another avenue for exploration could be to the altered attentional process to pain-related information due to prolong exposure to chronic pain and pain management treatment from the hospital among chronic pain patients recruited from the hospital compared to community sample or college sample with chronic pain who have not been treated for their chronic pain in the hospital.

The present study did not find data to support the threat-interpretation model (Todd et al., 2015). The present study found that chronic pain patients who endorsed high levels of pain catastrophizing initially preferred neutral facial expressions whereas those with low pain catastrophizing initially preferred pain facial expressions. According to the threat-interpretation model (Todd et al., 2015), individuals who interpreted stimuli as threatening tend to be drawn to threat-related information during the early stage of attention. The opposite attentional pattern observed in the present study may imply that chronic pain patients who have been treated for their chronic pain in the hospital and endorsed high level of pain catastrophizing initially avoid pain-related information in order to manage the negative emotions associated with pain-related stimuli. This result may also be explained by either the altered attentional process for the chronic pain hospital-treated patients or low self-referential values for pain facial expression stimuli compared to pain word stimuli. However, interpretation of these results should be made with caution since the results were not significant.

Moreover, attentional avoidance from pain-related information was also not observed for the high pain catastrophizing group during the later stage of attention. A previous study (Lee et al., 2019) that utilized similar research methods also found non-significant effects of pain catastrophizing on attentional bias to pain facial expressions. As discussed earlier, this result may be due to the fact that chronic pain patients’ experience of outpatient hospital-based treatment alters their attentional patterns to pain facial expressions. Another possibility may be due to the fact that more than half of the chronic pain participants in the present study have suffered from chronic pain associated with autoimmune diseases. Autoimmune diseases can cause pain on multiple parts of the body simultaneously and the pain locations may change time to time. Therefore their pain experiences may not be same as chronic pain patients with musculoskeletal disorders or disorders with localized chronic pain. For chronic pain patients with autoimmune diseases, unpredictability, lethargy or uncomfortable sensations on different part of their body may be more disabling and worrisome as those symptoms directly affects their daily functions rather than pain sensations. Therefore, they may not react sensitively to pain stimuli and interpret pain stimuli as threatening in the same manner as chronic pain patients with musculoskeletal disorders. So, for those individuals, interpreting ambiguous bodily sensations as threatening and catastrophizing during the early stage of attention can determine attentional engagement to those stimuli during the later stage of attention (Van Ryckeghem et al., 2019). Future studies need to compare attentional and interpretational biases between chronic pain patients with different types of chronic pain conditions (e.g., musculoskeletal disorders vs. autoimmune diseases) and compare attentional biases toward different types of stimuli including ambiguous sensorimotor stimuli, pictures of body part relevant to the pain location as well as symbolic pain stimuli.

The third possibility for non-significant results of the present study (i.e., non-significant effect of pain catastrophizing on attentional patterns to pain stimuli among chronic pain individuals) is the complex and multifaceted nature of pain catastrophizing construct (Schütze et al., 2017, 2020). This concept includes the emotional construct (i.e., helplessness), the cognitive process construct (i.e., rumination), and the cognitive content construct (i.e., magnification). Schütze et al. (2017) also argued that pain catastrophizing is a more “state-like,” not “trait-like” construct that can change depending on internal and external triggers, pain levels, metacognition about rumination (rumination can help to solve problems vs. rumination is uncontrollable and harmful to manage pain), and coping activities. Future studies need to utilize multiple assessments of pain catastrophizing in order to investigate the effects of pain catastrophizing on attentional bias to pain stimuli among chronic pain patients.

Lastly, the results of the present study suggested that chronic pain patients with high catastrophizing levels reported greater disability with pain and depression even though their chronic pain levels on past 3 months were not significantly higher than low pain catastrophizing group. This is interesting result as current study did not support the evidence for the attentional engagement to pain stimuli among chronic pain patients, particularly those with high level of pain catastrophizing. The results of the present study indicated that pain catastrophizing level may not play a role in the increase of attention to pain facial expressions but may play a role in the increase of catastrophic interpretations of pain and avoidance of activities, which can worsen both depression and disability associated with chronic pain. Although generally chronic pain patients did not over-attend to pain stimuli because they did not perceive pain stimuli as threatening, those who catastrophize may interpret pain stimuli as threatening and as a result, experience increased pain-related disability as well as psychological distress by avoiding activities due to hypervigilance to their bodily sensations. Clinicians should be mindful that interventions to redirect or distract the patient’s attention from pain stimuli may be less effective in decreasing pain disability and psychological distress of chronic pain patients, particularly among chronic pain patients with autoimmune diseases who endorse high pain catastrophizing levels. Psychological interventions that target decreasing catastrophic interpretation of pain-related information may be more effective. Furthermore, psychological interventions that consider different components of pain catastrophizing construct (i.e., emotional, cognitive content, and cognitive process) may be more helpful for chronic pain patients who endorse high levels of pain catastrophizing.

There are limitations for this study. First, sample size of the present study was limited to compare attentional patterns and the role of pain catastrophizing levels on attentional engagement to pain-related information across different types of chronic pain conditions. Additionally, the present study was cross-sectional study so the long-term effect of pain catastrophizing levels on attentional process to pain-related information was not able to be examined. Future studies should include a large number of chronic pain patients over time to investigate the role of pain catastrophizing on attentional patterns to pain-related information and long-term differences between chronic pain patients and healthy adults. Third, chronic pain groups were divided according to their scores of pain catastrophizing, and the statistical method utilized was a median split. Although researchers have raised concerns about using this method because it can increase type I errors and also make it harder to detect possible interaction effects, recent research (Iacobucci et al., 2015) has found, however, that the median split is a legitimate statistical method when independent variables are uncorrelated. Lastly, although the pain expression photo stimuli in the present study were validated in one of our previous studies (Lee et al., 2018) that utilized the same eye tracking methodology, the raters for those stimuli were either healthy adults or college-attending adults with chronic pain. Future studies need to address this limitation by validating pain expression photo stimuli with chronic pain patients and finding ways to measure how each pain photo was appraised as pain-related stimuli after the experiment is completed.

In spite of limitations, the present study can contribute to the field of attentional bias research in a number of ways. Despite extensive research on attentional bias toward pain stimuli for individuals with chronic pain, few studies investigated the time course for the effect of pain catastrophizing on chronic pain patients across patients with more broad range of chronic pain conditions including autoimmune diseases, and musculoskeletal disorders. Even though the present study did not find the significant effect of pain catastrophizing on attentional patterns to pain stimuli, we found pain catastrophizing plays a role for psychological health and life adjustment for the chronic pain patients. Results of the present study may stress the importance of investigating the interplay among attention, interpretation and psychological factors such as pain catastrophizing because cognitive bias to pain-related information can be adaptive or maladaptive depending on contexts (Van Ryckeghem et al., 2019).

## Data Availability Statement

The datasets generated for this study are available on request to the corresponding author.

## Ethics Statement

The studies involving human participants were reviewed and approved by the Institutional Review Board of Chung-Ang University Hospital (1711-002-267). The patients/participants provided their written informed consent to participate in this study.

## Author Contributions

JL involved in conception, acquisition of data, experimental design, data analysis, data interpretation, and manuscript development. J-HL was responsible for supervision, experimental design, data-analysis, interpretation, and manuscript development. S-WA involved in acquisition of data. AW involved data analysis, data interpretation, and manuscript development. All authors contributed to the article and approved the submitted version.

## Conflict of Interest

The authors declare that the research was conducted in the absence of any commercial or financial relationships that could be construed as a potential conflict of interest.
